# Frequency and levels of candidate endodontic pathogens in acute apical abscesses as compared to asymptomatic apical periodontitis

**DOI:** 10.1371/journal.pone.0190469

**Published:** 2018-01-02

**Authors:** Isabela N. Rôças, José F. Siqueira

**Affiliations:** Department of Endodontics and Molecular Microbiology Laboratory, Estácio de Sá University, Rio de Janeiro, RJ, Brazil; University of North Carolina at Chapel Hill, UNITED STATES

## Abstract

**Introduction:**

Acute apical abscess is caused by bacteria that leave the infected dental root canal to invade the periodontal tissues. Most species occurring in abscesses are also found in asymptomatic infections; therefore, the possibility exists that not only the presence of certain species but also their specific counts influence the appearance of symptoms. This molecular study compared the frequency and levels of several candidate endodontic pathogens in teeth with acute apical abscesses and asymptomatic apical periodontitis.

**Methods:**

Samples were taken from the root canals of teeth with asymptomatic apical periodontitis (n = 73) and by aspiration of purulent exudate from acute abscesses (n = 55). DNA was extracted from samples and bacterial identifications were performed by a closed-ended semi-quantitative reverse-capture checkerboard approach targeting 40 bacterial species/phylotypes.

**Results:**

Bacterial DNA was detected in all cases. In abscesses, the most prevalent taxa were *Fusobacterium nucleatum* (60%), *Porphyromonas endodontalis* (53%), *Parvimonas micra* (51%), and *Streptococcus* species (45%). The most frequently detected taxa in asymptomatic teeth were *P*. *endodontalis* (63%), *Dialister invisus* (58%), *Olsenella uli* (56%), and *F*. *nucleatum* (51%). None of the targeted taxa were significantly associated with abscesses when only mere presence was evaluated (p>0.05). However, semi-quantitative data demonstrated that *P*. *endodontalis*, *Prevotella baroniae*, *Treponema denticola* and *Streptococcus* species were significantly more frequent at levels >10^5^ in abscesses than in asymptomatic cases (p<0.05).

**Conclusion:**

None of the target species/phylotypes were associated with abscesses in terms of frequency. However, some taxa were significantly found in higher levels in abscesses. Presence of a potentially virulent pathogen in high counts may increase the collective pathogenicity of the bacterial community and give rise to symptoms.

## Introduction

Apical periodontitis is a very common disease caused by bacterial infection of the dental root canal [1]. Intracanal bacteria are usually organized in biofilm structures, dominated by obligate anaerobic species forming a mixed community [2, 3]. Apical periodontitis may be asymptomatic or symptomatic. The acute apical abscess is the most dramatic symptomatic form of the disease and is mostly characterized by severe pain and swelling. Sometimes, the endodontic abscess can spread to fascial spaces of the head and neck and result in complications, including systemic manifestations, such as fever, lymphadenopathy, and malaise [4].

Over the years, research has focused on finding the specific bacterial species associated with symptomatic disease, including abscesses. While some Gram-negative anaerobic bacteria have been found in association with symptoms of endodontic infections [5–9], the same species have been encountered in similar frequencies in teeth with no symptoms whatsoever [10–15]. Thus, it has been suggested that, in addition to the presence of certain species, the development of symptoms of acute infection is a result of the interplay of diverse bacterial and host factors, including counts of the specific taxa occurring in the infected site [1, 4].

Culture-independent molecular studies have been widely used to unearth the diversity of primary endodontic infections [16]. These methods have the advantage over culture of being able to detect and identify species that are difficult or even impossible to cultivate [17]. Even identification of cultivable species can be more reliable when molecular methods are used [18]. Although numerous molecular studies have been published evaluating the endodontic microbiota, only a few have compared symptomatic and asymptomatic infections. Overal, studies have shown that the structure of endodontic communities (types of species and their levels) differs between symptomatic and asymptomatic teeth [19–21]. Therefore, an important factor that may influence the pathogenicity of the community is the level of certain key species.

Specific infectious load relates to the counts of certain pathogenic species in the infected site. The levels of specific virulent species or strains is certainly of utmost importance for community pathogenicity. This factor may help explain why several species have been found in similar prevalence in both symptomatic and asymptomatic infections. The large majority of culture-dependent and culture-independent studies have evaluated only the prevalence of species (number of positive cases), with no counting efforts. If some species are present in higher numbers in symptomatic cases than in cases with no symptoms, then an important association is disclosed. For instance, a study identified *Tannerella forsythia* in similar prevalence but in significantly higher levels in symptomatic than in asymptomatic teeth [22].

This study was undertaken to evaluate the frequency and levels of 40 oral bacterial taxa, most of them regarded as candidate endodontic pathogens, in samples taken from acute apical abscesses and teeth with asymptomatic apical periodontitis using the semi-quantitative reverse capture-checkerboard DNA-DNA hybridization approach.

## Material and methods

### Subjects and sample taking procedures

Samples were taken from 133 patients (85 males and 48 females; mean age of 42 years, ranging from 16 to 75 years) who had been referred for root canal treatment or emergency treatment to the Department of Endodontics, Estácio de Sá University, Rio de Janeiro, RJ, Brazil. Each patient contributed one single-rooted tooth. All included teeth showed carious lesions, necrotic pulps and radiographic evidence of periapical bone destruction. Seventy-eight cases were diagnosed as asymptomatic apical periodontitis and the other 55 as acute apical abscess. Patients with abscesses presented with pain and localized or diffuse swellings and no apparent communication of the abscess with the oral mucosa or skin surface. None of the included teeth had periodontal pockets deeper than 4 mm. Medical history of the patients was non-contributory. The study protocol was approved by the Ethics Committee of the Estácio de Sá University and written informed consent was obtained from all individuals or their parents.

Samples from asymptomatic teeth were taken from the necrotic root canals under strict aseptic conditions. After oral rinse with 0.12% chlorhexidine, the supragingival biofilm was removed by scaling and cleansing with pumice. Caries and/or coronal restorations were removed with sterile high-speed burs under sterile saline irrigation. Following rubber dam isolation, the operative field, including the tooth, clamp and surrounding dam, was cleaned by using 3% hydrogen peroxide and then disinfected with 2.5% NaOCl. Access preparation was completed with sterile burs under sterile saline irrigation, and the operative field, this time also including the pulp chamber, was once again cleaned and disinfected as above. Ten percent sodium thiosulfate was used to neutralized the residual NaOCl and sterility control samples were collected to evaluated the effectiveness of the disinfecting approach. These samples were taken from the internal surface of the cavosurface angle of the access cavity by using sterile paper points, as described elsewhere [23, 24]. The area sampled was the one from the access cavity walls where the paper points used later for taking root canal samples might accidentally touch. Paper points were transferred aseptically to a tube containing Tris-EDTA (TE) buffer (10mM Tris HCl, 1mM EDTA, pH = 7.6) and immediately frozen at -20°C. For inclusion of the tooth in the study, sterility control samples had to be negative for bacterial DNA in the same polymerase chain reaction (PCR) assay as used for checkerboard analysis (see below). Accordingly, 5 samples were excluded from the study.

The canal was filled with sodium thiosulfate, a small hand instrument was placed up to 1 mm short of the root apex as radiographically determined and worked in gentle circumferential filing motion. The canal was then sampled by using sterile paper points up to that level. Each paper point was left in the canal for about 1 min to absorb the canal content. Care was taken to avoid touching the walls of the access cavity with the paper points during sampling. Paper points were immediately placed in tubes containing TE buffer and frozen at -20°C.

Samples from acute abscesses were taken by aspiration of pus from the swollen mucosa over each abscess. After oral rinse with 0.12% chlorhexidine followed by disinfection of the overlying mucosa with 2% chlorhexidine, a sterile disposable syringe was used to aspirate the purulent exudate through the swollen mucosa. Pus was immediately injected into tubes containing TE buffer and frozen at -20^°^C.

### Semi-quantitative reverse-capture checkerboard assay

Clinical samples were thawed to 37°C for 10 min and agitated for 1 min. DNA was extracted using the QIAamp DNA Mini Kit (Qiagen, Valencia, CA, USA), following the manufacturer’s directions. DNA from a panel of several oral bacterial species was also prepared to serve as controls [13, 25].

The reverse-capture checkerboard assay was used to identify 40 oral bacterial taxa. Whole-genomic DNA extracts from clinical samples were used as templates in a two-step 16S rRNA gene-based PCR protocol. In the first one, a practically full-length 16S rRNA gene fragment was amplified using universal primers 8f and 1492r [26–28] and 5- μl aliquots of DNA extracts. In the second step, PCR products from the 1^st^ reaction were used as template to run 2 sets of partial 16S rRNA gene amplification, one using primers digoxigenin-8f and 519r and the other using primers digoxigenin-515f and 1492r [15]. Thus, 2 different fragments were obtained for each sample; these fragments together comprise approximately the entire length of the 16S rRNA gene. Because 2 different checkerboard runs had to be performed for each sample (30 different probes per run), the 1^st^ PCR product served as template for two sets of labeled hemi-nested amplification with the 2 primer pairs. Aliquots of 1 μl of the 1^st^ PCR product were used in each set of second round amplification.

PCR was carried out in a 50- μl reaction mixture containing 1 μM of each primer, 5 μl of 10 × PCR buffer (Thermo Fisher Scientific, Foster City, CA, USA), 3 mM MgCl_2_, 2 U of *Taq* DNA polymerase (Thermo Fisher Scientific) and 0.2 mM of each deoxyribonucleoside triphosphate (Thermo Fisher Scientific). Negative controls consisted of sterile ultrapure water instead of sample and were included with each batch of samples analyzed.

PCR cycling conditions for the 1^st^ reaction using primers 8f/1492r was: 95^°^C/1min, 26 cycles at 94^°^C/45s, 50^°^C/45s, and 72^°^C/1.5min, and 72^°^C/20min. For the 2^nd^ round of amplification using primers digoxigenin-8f/519r or digoxigenin-515f/1492r: 95^°^C/5min, 28 cycles at 94^°^C/30s, 55^°^C/1min, and 72^°^C/1.5min, and 72^°^C/20min. Presence of PCR amplification products was evaluated after agarose gel electrophoresis using ultraviolet transillumination.

Labeled PCR products from the two reactions were mixed using equal proportions of each and used in the checkerboard assay to determine the presence and levels of 40 bacterial taxa. 16S rRNA gene-based probes were as described and validated elsewhere [15, 28, 29]. Probes were randomly distributed along two different membranes. In addition to the taxon-specific probes, 2 universal probes were included in each membrane to serve as controls. Two lanes in the membrane contained DNA standards at the concentration of 10^5^ and 10^6^ cells, which were treated the same way as the clinical samples.

The Minislot-30 and Miniblotter-45 system (Immunetics, Cambridge, MA, USA) was used to run the reverse-capture checkerboard assay. First, 100 pmol of probe in TE buffer (pH 8.0) were introduced into the horizontal wells of the Minislot apparatus, and crosslinked to the Hybond- N+ nylon membrane (AmershamPharmacia Biotech, Buckinghamshire, England) by ultraviolet irradiation using a Stratalinker 1800 (Stratagene, La Jolla, CA, USA) on autocrosslink position. Each probe has a polythymidine tail that preferentially crosslinks to the nylon and leaves the specific probe available for hybridization. The membrane was prehybridized at 55^°^C for one hour. Next, the labeled PCR products mixed with 55^°^C preheated hybridization solution were denatured at 95°C/5min and loaded on the membrane using the Miniblotter apparatus. Hybridization was carried out at 54^°^C for 2 hours.

After hybridization, the membrane was washed and blocked in a buffer with casein. Next, it was incubated in antidigoxigenin antibody conjugated with alkaline phosphatase (Roche Molecular Biochemicals, Mannheim, Germany) and ultra-sensitive chemiluminescent substrate CDP Star (Roche Molecular Biochemicals). Finally, a square of radiographic film was exposed to the membrane in a cassette for 10 minutes in order to detect the hybrids.

### Data analysis

Prevalence of the target bacterial taxa was recorded. For determination of levels, a semi-quantitative analysis was conducted as follows. Chemiluminescent signals were evaluated using the ImageJ 1.50d software (National Institutes of Health, Bethesda, MD, USA) and converted into counts by comparison with standards at known concentrations run on each membrane. Because of the recognized difficulties in inferring absolute counts for PCR-amplified samples and because estimates had to be made for counting as-yet-uncultivated phylotypes or culture-difficult species, counts were considered as heavy (a signal stronger than the 10^5^ standard) or mild (a signal weaker than the 10^5^ standard). No signal means absence of the target taxon or presence in numbers below the method´s detection threshold, which was approximately 10^3^.

The chi-square test or the two-tailed Fisher’s exact test was used to compare the prevalence and levels of the detected taxa in abscesses and asymptomatic cases. Association of patients’ gender and age with symptoms was evaluated by the chi-square test and the Student t test, respectively. Statistical analyses were conducted using the Statistica software (version 8.0, StatSoft, Tulsa, OK, USA). Significance level was set at p<0.05.

## Results

Bacterial DNA was detected in all samples by PCR using universal 16S rRNA gene primers. All oligonucleotide probes tested were reactive with at least 9 or more clinical samples. Overall, the most prevalent taxa were *Porphyromonas endodontalis* (75/128; 59%), *Fusobacterium nucleatum* (70/128; 55%), *Dialister invisus* (64/128; 50%), *Olsenella uli* (63/128; 49%), *Parvimonas micra* (61/128; 48%), and *Prevotella baroniae* (52/128; 41%).

**Fig 1:**
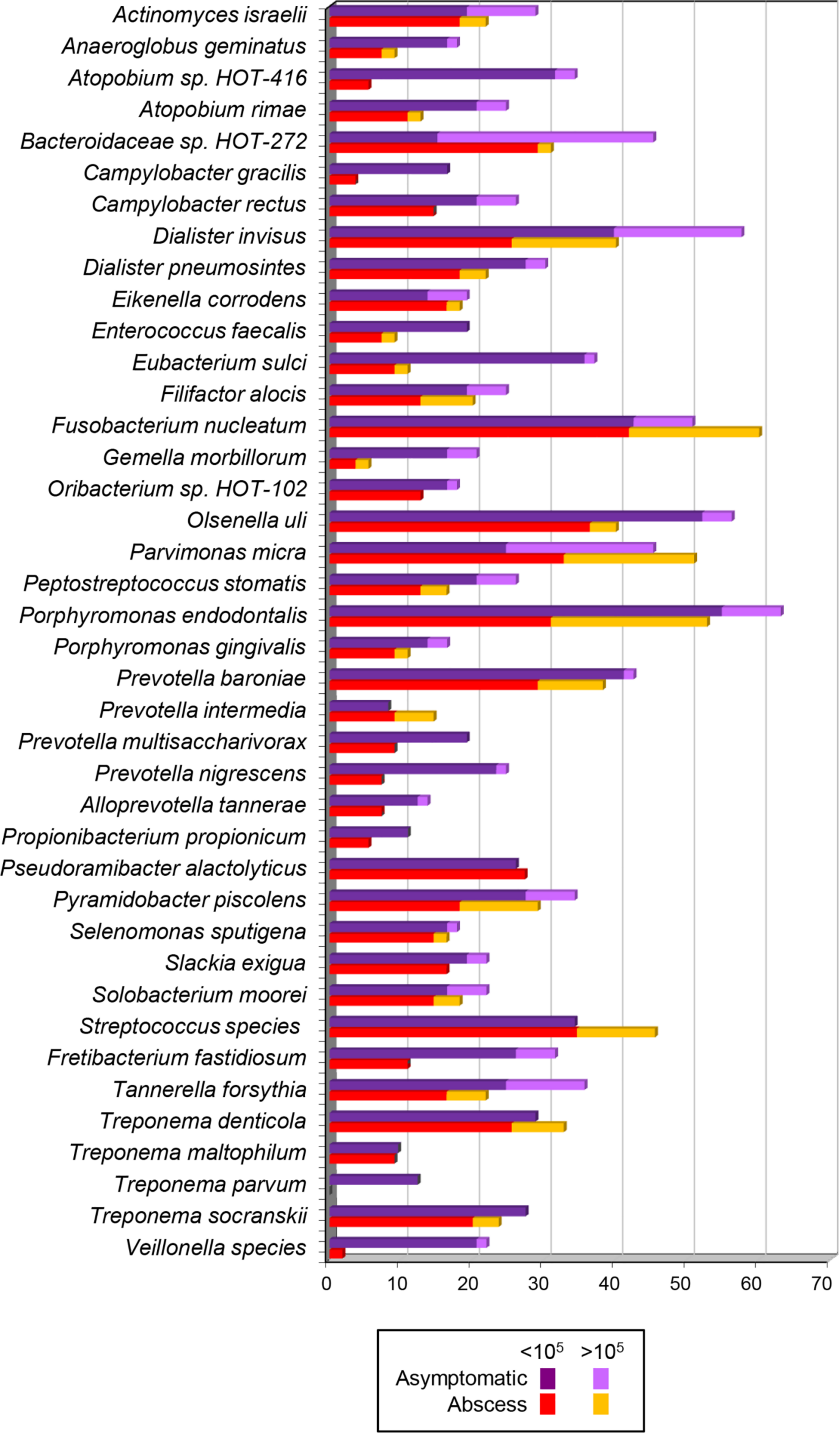
Stacked bar chart of frequency of detection and levels of bacterial taxa in samples from teeth with acute apical abscess or asymptomatic apical periodontitis. Total length of each bar stack indicates percentage of positive samples. Different colors within each bar indicate percentage of samples containing each level range (< or > 10^5^ cells).

All the target taxa occurred in canals of asymptomatic teeth in at least 6 cases. All samples were positive for at least 2 taxon-specific probes. The most frequently detected taxa in asymptomatic teeth were *P*. *endodontalis* (46/73; 63%), *D*. *invisus* (42/73; 58%), *O*. *uli* (41/73; 56%), and *F*. *nucleatum* (37/73; 51%)(Fig 1).

All but one taxa (*Treponema parvum*) were detected in at least one abscess sample. All samples were positive for at least 3 taxon-specific probes. The most prevalent taxa in abscesses were *F*. *nucleatum* (33/55; 60%), *P*. *endodontalis* (29/55; 53%), *P*. *micra* (28/55; 51%), and *Streptococcus* species (25/55; 45%)(Fig 1).

Frequency (presence/absence) data revealed that none of the targeted taxa were significantly associated with symptomatic cases (p>0.05). Actually, the following taxa were significanty more detected in asymptomatic teeth: *Atopobium* sp. HOT416, *Campylobacter gracilis*, *Eubacterium sulci*, *Gemella morbillorum*, *Prevotella nigrescens*, *Fretibacterium fastidiosum*, *T*. *parvum* and *Veillonella* species (p<0.05).

Semi-quantitative data demonstrated that only 4 of the target taxa were significantly more prevalent at levels >10^5^ in symptomatic cases than in asymptomatic cases (p<0.05); *P*. *endodontalis*, *P*. *baroniae*, *Streptococcus* species and *Treponema denticola*.

Sex and age showed no significant association with symptoms (p>0.05).

## Discussion

An area of intense interest in endodontic microbiology is the search for pathogens specifically involved with symptom causation. Although the microbiota of symptomatic endodontic infections including abscesses have been deciphered by culture and molecular methods [4, 30], no specific single species or even groups of species have been definitely proven as the main culprits. Evidence mounted over the years has suggested that factors other than the mere presence of a potentially pathogenic species may determine the severity of the infection and influence the appearance of symptoms [1]. Endodontic infections are characterized by multispecies biofilm communities and the disease outcome is dependent upon the collective pathogenicity of these communities [31]. Community pathogenicity is conceivably a result of several factors, including the type of species and virulence of the clonal types present, synergistic interactions between community members, and the numbers (counts) of each species composing the community [4]. Findings from the present study indicated that some species/phylotypes were indeed significantly more encountered in asymptomatic cases, and no one was linked to symptoms in terms of frequency. However, some taxa occurred in significantly higher counts in abscesses.

Many studies have found some candidate endodontic pathogens in positive association with symptoms [5–9]. However, others failed to confirm these findings, by detecting the same species in similar or even higher prevalence in asymptomatic teeth [10–12, 14, 15]. This was also observed in the present study when only frequency data were analyzed. Some species such as *F*. *nucleatum*, *O*. *uli*, *D*. *invisus*, and *P*. *micra* were highly prevalent in abscesses, but they also occurred in similarly high frequencies in asymptomatic cases. The large majority of previous culture and molecular studies focused only on prevalence with no attempt to quantify the bacterial taxa composing the endodontic mixed consortium.

In a cross-sectional study, a species that is found in both asymptomatic and symptomatic infections can be regarded as having no association with symptoms. Nevertheless, association may pass unnoticed if evaluation is not quantitative. This was clearly demonstrated in the present study. Although *P*. *endodontalis*, *P*. *baroniae*, *Streptococcus* species and *T*. *denticola* were found in statistically similar frequencies in samples from both abscesses and asymptomatic disease, these taxa occurred in levels significantly higher in the former. This suggests that presence of these taxa in high levels may be important to increase the bacterial community collective pathogenicity and induce symptoms.

*P*. *endodontalis* was for the first time isolated from a root canal in association with symptoms [32] and since then it has been frequently detected in abscesses of endodontic origin by both culture and molecular methods [33–36]. This species can cause purulent infections in animals, especially in mixed cultures [37, 38]. The ability to induce abscesses may be related to activation of pro-inflammatory cytokines by *P*. *endodontalis* lipopolysaccharide [39]. Other virulence factors of *P*. *endodontalis* include capsule, outer membrane proteins, proteinases and cytotoxic metabolic products [38].

*P*. *baroniae* was first detected by a culture-independent approach in endodontic abscesses and provisionally named clone PUS9.180 [40]. It was later cultivated and the species name proposed [41]. It has also been commonly detected in endodontic infections, including abscesses [19, 36, 42, 43].

*Streptococcus* species have been frequently encountered in different types of endodontic infections, including abscesses [13, 42, 44, 45]. Streptococci most commonly found in odontogenic abscesses belong to the *anginosus* group (*S*. *anginosus*, *S*. *constellatus* and *S*. *intermedius*)[13, 42, 45]. Indeed, this group has been demonstrated to induce abscesses in animal models [37, 46]. Because other *Streptococcus* species can also be found in endodontic infections [13, 42, 45], a probe for the genus was used in this study. Further studies should elaborate more on the diversity and levels of *Streptococcus* species/phylotypes associated with abscesses.

*T*. *denticola* is an important periodontal pathogen that was first detected in endodontic infections by molecular methods [12, 47]. Treponemes had been suggested as important causative agents of endodontic abscesses by the end of the 19^th^ century [48], but only after the advent of culture-independent methods they have been identified in root canal and abscess samples [12, 47, 49]. *T*. *denticola* is the most prevalent treponeme in most studies of endodontic infections and has been found in high frequencies in abscesses [50–53]. It can cause severe and disseminating pulp infections in immunocompetent and severe combined immunodeficient mice [54]. Although its prevalence in abscesses was not high in the present study, when present this treponeme commonly occurred in high levels.

A semi-quantitative reverse-capture checkerboard approach was used to identify bacterial taxa at levels below or above 10^5^, a level considered herein as a cut-off for mild or heavy infection. The checkerboard approach permitted to screen a large number of samples for the presence of several bacterial taxa and the semi-quantitative data revealed some specific species/phylotypes that should be used as targets for further studies using more accurate quantitative analyses, as for instance the real-time PCR for absolute counts. High-throughput sequencing approaches have been widely used for a deep coverage of bacterial identification in endodontic infections [20, 55–58]. However, they provide information on bacterial prevalence and relative abundance; while the latter is important to infer dominance in the community, a pathogenic role cannot be directly inferred. For instance, if the overall bacterial counts are low, even a dominant species may not be in levels sufficient to be virulent.

The species/phylotypes that were selected to be targeted in this study have been regarded as candidate endodontic pathogens based on their prevalence and abundance in primary endodontic infections [3]. Prevalence of different species/phylotypes is expected to vary from study to study, but the large majority of evaluations of endodontic infections in diverse geographic locations have virtually always included most of the species/phylotypes evaluated herein, especially *F*. *nucleatum*, *Dialister* species, *P*. *endodontalis*, *P*. *micra*, *Streptococcus* species, *Prevotella* species, and *Treponema* species [3]. All these taxa were frequently detected in both abscesses and asymptomatic apical periodontitis, confirming their status as candidate endodontic pathogens.

Samples from the two conditions were taken from different sites, i.e., the root canal for asymptomatic cases and the periapical tissues for abscesses. In abscesses, bacterial infection has already spread to the periapical tissues and an extraradicular infection occurs in a continuum with the intraradicular component. We opted for taking samples from the periapical purulent exudates, because bacteria located in this area are in the forefront of the infectious process and as such are those directly involved with abscess formation. In these cases, collecting samples from the root canals instead of the periapical tissues would also include bacterial species/phylotypes located in the most coronal areas of the canal, which are not expected to be directly pariticipating in the periapical acute infectious process. In asymptomatic cases, infection is usually restricted to the root canal; bacteria involved directly with disease pathogenesis are expected to be those located in the apical part of the canal system and an extraradicular infection is usually absent [2]. In the clinical situation, there is no way to take samples exclusively from the apical canal in these cases. Only by using extracted teeth and cryopulverizing the apical root segment could one evaluate the apical canal microbiota of asymptomatic teeth [57]. One should take into account these differences when interpreting the results from the present study, because the possibility exists that prevalence/counts of some taxa may have been underestimated in abscesses (by precluding bacteria in the apical canal system) and/or overestimated in the asymptomatic teeth (by including bacteria in the coronal parts of the canal).

In conclusion, the present study demonstrated that none of the target species/phylotypes were associated with abscesses in terms of frequency. However, when levels were evaluated, some taxa were significantly found in higher counts in abscesses. This helps explain the previous and current non-quantitative findings showing no significant association of specific bacterial taxa with symptoms. *P*. *endodontalis*, *P*. *baroniae*, *Streptococcus* species and *T*. *denticola* were found in both abscesses and asymptomatic teeth in similar prevalence, but occurred in higher levels in the former. Along wirh other factors [4], presence of a potentially virulent pathogen in high counts may increase the virulence of the whole community and influence the appearance of symptoms of endodontic infections.
